# *QuickStats: *Percentage* of Office-Based Physicians Who Had Telephone or Internet/Email Consults with Patients^†^ — National Ambulatory Medical Care Survey, United States, 2018 and 2020^§^

**DOI:** 10.15585/mmwr.mm7113a4

**Published:** 2022-04-01

**Figure:**
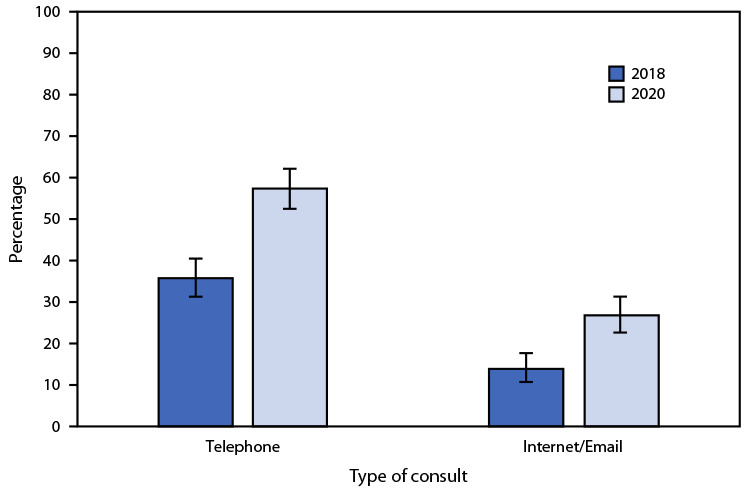
The percentage of office-based physicians who reported having telephone consults with patients during their last normal week of practice increased from 35.8% in 2018 to 57.4% in 2020. The percentage who reported having Internet/email consults with patients also increased from 13.9% in 2018 to 26.8% in 2020. In both years, physicians were more likely to report having telephone than Internet/email consults.

**Source:** National Center for Health Statistics, National Ambulatory Medical Care Survey, 2018 and 2020. https://www.cdc.gov/nchs/ahcd/about_ahcd.htm

